# Formulation of Environmentally Safe Graffiti Remover Containing Esterified Plant Oils and Sugar Surfactant

**DOI:** 10.3390/molecules26154706

**Published:** 2021-08-03

**Authors:** Marcin Bartman, Sebastian Balicki, Kazimiera A. Wilk

**Affiliations:** Department of Engineering and Technology of Chemical Processes, Wrocław University of Science and Technology, Wybrzeże Wyspiańskiego 27, 50-370 Wrocław, Poland; marcin.bartman@pwr.edu.pl (M.B.); sebastian.balicki@pwr.edu.pl (S.B.)

**Keywords:** eco-friendly graffiti remover, esterified plant oils, sugar surfactants, green solvents, environmentally friendly, formulation optimization, response surface methodology

## Abstract

The removal of graffiti or over-painting requires special attention in order to not induce the surface destruction but to also address all of the important eco-compatibility concerns. Because of the necessity to avoid the use of volatile and toxic petroleum-based solvents that are common in cleaning formulations, much attention has recently been paid to the design of a variety of sustainable formulations that are based on biodegradable raw materials. In the present contribution we propose a new approach to graffiti cleaning formulations that are composed of newly synthesized green solvents such as esterified plant oils, i.e., rapeseed oil (RO), sunflower oil (SO), or used cooking oil (UCO), ethyl lactate (EL), and alkylpolyglucosides (APGs) as surfactants. Oil PEG-8 ester solvents were synthesized through the direct esterification/transesterification of these oils using monobutyltin(IV) tris(2-ethylhexanoate) and titanium(IV) butoxide catalysts under mild process conditions. The most efficient formulations, determined by optimization through the response surface methodology (RSM) was more effective in comparison to the reference solvents such as the so-called Nitro solvent (denoting a mixture of toluene and acetone) and petroleum ether. Additionally, the optimal product was found to be effective in removing graffiti from glass, metal, or sandstone surfaces under open-field conditions in the city of Wrocław. The performed studies could be an invaluable tool for developing future green formulations for graffiti removal.

## 1. Introduction

The presence of graffiti on buildings facades, train stations, trains, buses, and street furniture including traffic lights and garbage bins is a problem that affects many inhabitants in metropolitan or urban areas and needs certain attention [1,2,3]. For thousands of years, graffiti comprised a form of artistic or revolutionary expression whose visual appearance and types of inks along with its motivational origins have nowadays transformed significantly. By the majority of society, it is now considered as sort of vandalism since it does not impart a message to the spectators and only serves as a means of communication between the painters [4]. It has to be emphasized that graffiti removal constitutes an expensive process, and inadequate cleaning methods might either damage a given surface or might not be effective enough to maintain the required aesthetic impacts of the buildings. This is why a variety of preventive and curative methods have been developed to handle the aforementioned problems [5,6].

According to the type of graffiti and the affected surface, there are the following two main classes of removal techniques: chemical and physical [7]. The most frequently applied approach comprises chemical-based cleaning processes which can be performed with organic solvents or solvent mixtures, usually supported by some mechanical action [7]. Extremely appealing in these aspects are the combinations of chemical cleaning solutions with the use of a hot water jet. Sometimes, in addition to the chemical methodology, it is satisfactory to wash the surfaces composed of non-porous materials with a cloth dampened with hot water [8]. Alternatively, physical methodologies can be implemented that include traditional tools, such as scalpels, abrasive media, and water or sand blasting, and more innovative approaches, such as laser ablation, ultrasonication, plasma spray, and biological cleaning [9,10,11,12,13]. Generally, it should be remembered that the fundamental requirement of cleaning—a crucial task aiming to restore original aesthetic appearance—is to be selective and non-invasive for the removal of the original components of objects or buildings. Chemical attempts that employ chemical solutions that are responsible for paint dissolution and extraction are treated as a traditional technique for graffiti removal [11,13]. Organic solvents such as methylene dichloride and acetone or alkali caustic removers are traditionally applied for a variety of surfaces [7,10,14]. Mixtures of organic solvents based on alcohols (ethanol, propyl, isopropyl, butyl, isobutyl, isoamyl, cyclohexanol, etc.), esters (butyl acetate, ethyl acetate, etc.), ketones (acetone, methyl ethyl ketone, etc.), and aromatic hydrocarbons (toluene, xylene, etc.) mixed in various proportions present a broad performance in graffiti cleaning in washing dirt or/and adherent deposits [15]. However, Weaver et al. [16] noted that some chemical solvents may permanently discolor or stain the building surface, and in addition, the remaining paint may become more difficult to remove. In fact, common solvents are principally volatile organic compounds and are harmful to both human health and the environment [17]. For humans, their inhalation or absorption through the skin can have harmful effects, such as skin and eye irritation, nausea, and dizziness, while in the case of long contact periods, they may cause damages to the liver, kidneys, and central nervous system [18]. Because of environmental and safety protections laws (REACH/CLP Regulation No. 1272/2008) and green chemistry and sustainable development, finding alternative solvents to petroleum-derived ones has become a major challenge for chemists [19].

The selection of the ideal solvent for graffiti removal purposes is a compromise between cleaning procedure efficiency and environmental and human protection [20]. The term “green solvents” no longer only refers to a series of organic solvents and supercritical CO_2_, but also to a variety of ionic liquids, switchable solvents and deep eutectic solvents, liquefied gases, supercritical fluids, and “bio-based solvents” that have been successfully applied according to the recent literature [20,21]. Solvents derived from renewable feedstock, i.e., obtained from biomass such as cellulose and starch, are considered as a relatively new class of reagents that meet the twelve green chemistry principles and the principles of sustainable development [22,23,24]. Therefore, for the development of eco-friendly graffiti removers, some topics, including the reduction/elimination of high toxicity reagents, decreasing the emission of gasses and vapors, and the reduction of energy consumption have recently gained significant importance [25,26]. The development of and the applications for novel and alternative solvents are of great importance in protecting cultural heritage stones, cleaning buildings, or the conservation of old icons or paints [15,27]. Much effort is needed to develop low-cost, effective technologies for the production of alternative solvents that could replace the typically applied toxic organic solvents. Among many outstanding examples of current highly efficient graffiti removers, there are highly retentive chemical hydrogels [28], nanofluids, such as micelles or microemulsions [28] as well as low-toxic solvent ternary mixtures followed by Nd:YAG laser irradiation [29].

**Figure 1:**
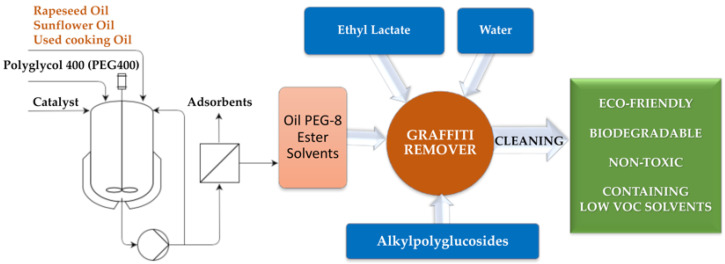
General approach of the performed studies. Oil PEG-8 ester solvents denote the following esterified oils (for the abbreviation see Table 1): MG-400-RO (derived from rapeseed oil), MG-400-SO (derived from sunflower oil), MG-400-UCO (derived from used cooking oil).

The proposal presented in this paper combines all of the abovementioned aspects, as shown in Figure 1. The main objective of the present study was to develop a new eco-friendly graffiti remover by formulating different types of mixtures comprising ecologically approved components, i.e., Oil PEG-8 ester solvents—esterified by polyglycols (PEG 400), selected plant oils (i.e., rapeseed oil (RO), sunflower oil (SO), or used cooking oil (UCO)) as well as ethyl lactate (EL) and alkylpolyglucosides C8/C10 (APGs) as surfactants and water, in an optimized manner.

The use of APGs—green and mild surfactants, which are often present in commercial cosmetic formulations and detergents [30]—to the best of our knowledge, constitutes one of the best attempts in graffiti remover use. Some profound examples with the use of alkyl glucoside to formulate nanostructured cleaning systems appeared in papers written by Baglioni et al. [2,31]. As recently observed by Krawczyk, the presence of APGs in a formulation can alter the minimal contact angle values on a given solid surface [32], thus resulting in the different wettability properties of the graffiti remover formulation. This property is especially crucial from the point of view of a material that should be cleaned or restored [33]. One possible graffiti removal method is the change in wettability of a particular solid material by using biosurfactants, natural surfactants, or their mixtures, which may be able to change the hydrophilic and hydrophobic characteristics of a discussed solid and its adsorption mechanisms [32]. Finally, the application of low-cost renewable bioresources that are disposable in large amounts makes it possible to develop a custom-designed product with a low environmental impact [34,35].

Therefore, we formulated natural biodegradable solvents that are capable for the efficient cleaning of different surfaces covered in graffiti paint that do not cause harm to the workers, environment, or equipment. The performance of the obtained graffiti removers was determined by their functional characteristics, i.e., viscosity and density, as well as efficiency measures such as runoff speed from flat surfaces and cleaning effectiveness in the function of time. If a product that did not have the most desired characteristics, the optimization of the multi-component mixture composition was elaborated upon. A variety of design models, such as full or fractional factorial models, as well as central composite or optimal group designs might be used in formulating an optimal graffiti remover [36,37]. In the current contribution, the d-optimal design method was used in order to determine the relation between formulation composition and its functional characteristics. The d-optimal design technique is considered to be one of the most effective tools for solving typical problems with mixture optimization where conventional designs do not apply. d-optimal design experimental matrices are typically not orthogonal, and the response factor (dependent variables) estimates are correlated. Through the usage of probability functions and value prediction, d-optimal models surpass conventional designs because they can be used even when the design space is constrained (where there are not perfectly convenient interactions between independent variables) and where too many experimental runs are not acceptable due to low resources and limited time for experimentation [38,39,40].

## 2. Results

### 2.1. Green Solvents as Esterified Natural Oil or Used Cooking Oil with PEG 400

The syntheses of plant Oil PEG-8 ester solvents (for the abbreviation see Table 1) were conducted to produce sample modified oil solvents through esterification, the latter creating eco-friendly graffiti removers. Esterification/transesterification was conducted according to the procedure described in the experimental portion of this work. In this experiment, the ratios of the reactants, the concentration of catalysts, the reaction time, and the reaction temperature and pressure were fixed, but the type of oil was changed. Table 1 shows the monitoring of process synthesis of Oil PEG-8 ester solvents using different types of oil. It can be seen that the conversion rate for free fatty acids was highly satisfactory when the metalloorganic Tin(Sn^4+^) and Titanium(Ti^4+^) compounds were used as the catalysts. The obtained results show that after an 8 h reaction, the mixture was stabilized and both the acid value, saponification value, hydroxyl value, and water content had not changed.

Table 2 summarizes the physical properties of the plant Oil PEG-8 ester solvents based on rapeseed oil (series denoted as MG-400-RO), sunflower oil (series denoted as MG-400-SO), and used cooking oil (series denoted as MG-400-UCO). The corresponding abbreviations for the obtained samples together with the formulation compositions and their controlled variables for each oil used as well as their functional characteristics are presented in Table 3. The results show that under identical reagents/catalyst ratios and the same reaction conditions, esterification/transesterification through the metalloorganic Tin(Sn^4+^) and Titanium(Ti^4+^) catalysts system affords similar Oil PEG-8 ester solvents and a rather reproducible molecular mass magnitude, i.e., the average molecular weights of M_n_ ranges from 550 M to 645 M (as show in Table 2). Fatty acid decarboxylation was not observed because the process temperature was low; therefore, the effect of the decomposition of fatty acids, which may be due to high temperature, does not occur [41]. The structure of RO, SO, and UCO consists of different tri-glyceride esters of saturated or unsaturated fatty acids and free fatty acids [42,43]. Therefore, the plant-based Oil PEG-8 solvents are mostly mixtures of monoester fatty acids with PEG-8, a mixture of the mono- or di-glycerides of fatty acids and polymers with low molecular weight and which could be derivatives of the dimers and trimers of unsaturated fatty acids, through polymerization and intermolecular Diels-Alder reactions [44,45].

### 2.2. Formulation of Eco-Friendly Graffiti Removers

There were sixty different formulations that were prepared and evaluated (as shown in Table 3). The formulations were identified with numbers (1–20) and the types of Oil PEG-8 ester solvents (MG-400-RO, MG-400-SO, MG-400-UCO). First of all, an assessment of the macroscopic characteristics of the formulations created was performed directly after preparation. The evaluation of the physical properties was performed immediately after the formulation was prepared. Thus, all of the eco-friendly graffiti removers (Formulas 1–20) (as shown in Table 3) based on rapeseed oil PEG-8 ester and used cooking oil PEG-8 ester that were prepared have a homogeneous appearance, while most of the formulations based on sunflower oil PEG-8 ester reveal a heterogeneous appearance. It needs to be pointed out that the appearance depended on the amount of water contained in the formulation. Typically, cloudy or white solutions were obtained when the water content was above 25% *w*/*w*. The macroscopic properties of graffiti removers depend on the fatty acid composition of the type of oil [46]. Thus, the appropriate combination of the concentration of oil PEG-8 ester, ethyl lactate, APGs, and water studied will lead to the formation of a desired graffiti remover formulation with satisfactory functional properties. 

### 2.3. The Speed of Runoff Eco-Friendly Graffiti Removers from Surface

The determinations of the speed of runoff characteristics were performed to evaluate the technical properties of the formulations and to assess which formulation is the most suited for application on the surface, as the residence time on the surface might affect the efficiency of the graffiti remover formulations. The tests were conducted according to the procedure described in the experimental portion of this work. Knowing the relationships between the formulation variables and its runoff speed from a surface is important because these effects can have an impact on graffiti paint removal efficiency [47]. Supplementary Table S1 presents the results of the runoff speed for an eco-friendly graffiti remover formulation from a given surface. The runoff speed test provides information on the runoff speed behavior of eco-friendly graffiti removers. These properties, which are specific to each type of formulation, can be modified in the presence of green solvents and sugar surfactant in the composition.

According to the runoff speed tests, the surface residence time of the eco-friendly graffiti removers depends on the appearance of the product. Therefore, the graffiti remover formulations that had emulsified due to the presence of a significant amount of water showed a twice or three times lower runoff speed than the homogeneous transparent or cloudy formulations.

The results of the performed tests on different reference products (petroleum ether and the Nitro solvent) in order to compare the runoff speed values showed that the surface runoff speed values of the reference products were comparable with each other (about 5 s). What was more relevant in the experiment was the observation that in comparison to the reference products, the developed eco-friendly graffiti remover with a homogeneous appearance was characterized by a residence time on a given surface that was three to four times higher than that the reference products. In other words, the tested formulation turned out to have a lower rate of speed than that of petroleum ether or the Nitro solvent. This, in turn, translates into a higher effectiveness in removing graffiti paint from various surfaces by means of the new green graffiti removal product.

### 2.4. The Effectiveness of Removing Graffiti Paints from the Flat Surface

**Figure 2:**
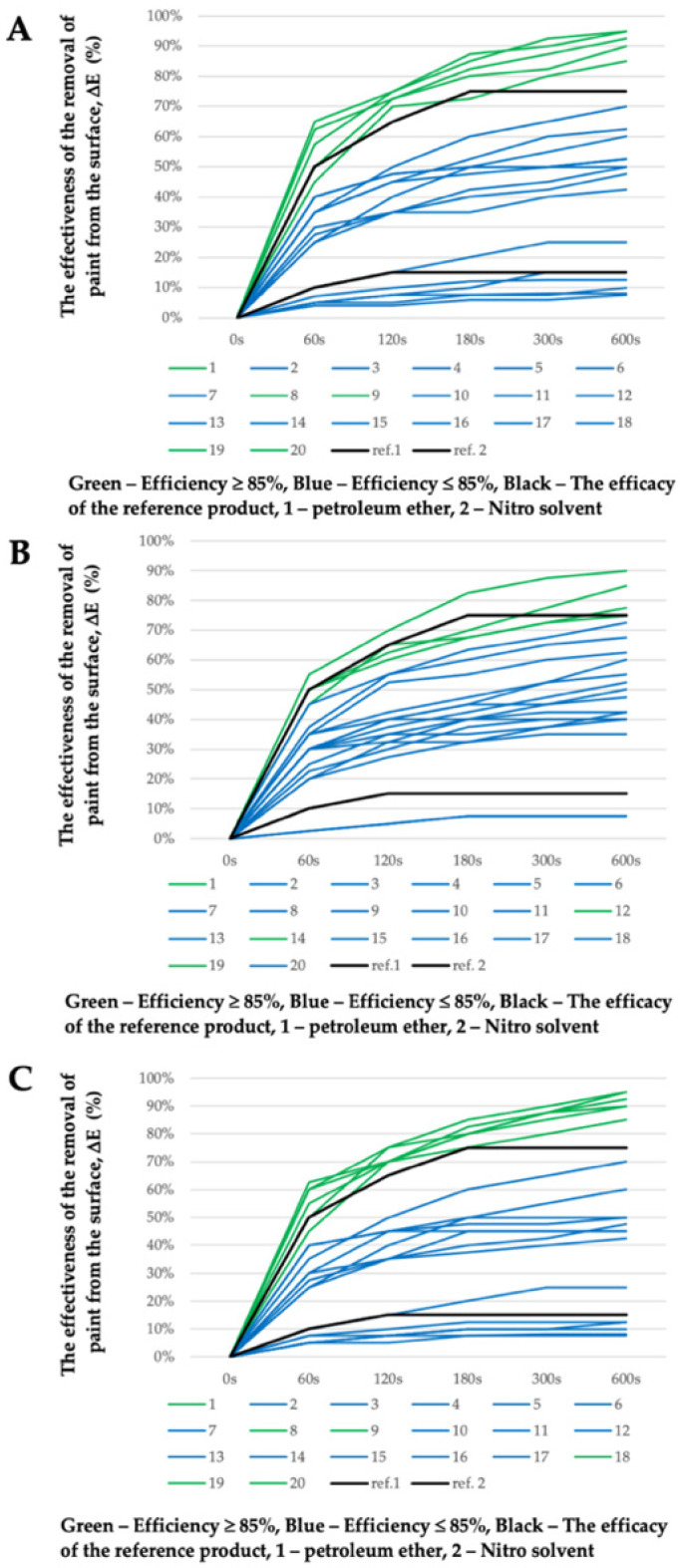
The effectiveness evaluation of the removal of graffiti paint from a surface (ΔE) in %; (**A**) graffiti remover formulations no. 1–20 based on MG-400-RO; (**B**) graffiti remover formulations no. 1–20 based on MG-400-SO; (**C**) graffiti remover formulations no. 1–20 based on MG-400-UCO.

The procedure described in the experimental section of this work was used to estimate the efficiency of removing graffiti paints from surfaces. Reference products such as petroleum ether and the Nitro solvent (i.e., a mixture of toluene and acetone) were used as cost–effectiveness thresholds. As it can be seen in Figure 2, the composition of an eco-friendly graffiti remover formulation greatly affects the formulation’s efficiency in removing graffiti paints from surfaces. Therefore, the effectiveness evaluations were made on the basis of the amount of graffiti remaining on the surface [48]. Considering the effectiveness evaluation of the cleaning properties of commercial products, it can be concluded that a ranking of the effectiveness for every formulation can be well established. The effectiveness of the removal of graffiti paint after 600 s can be sorted based on the reference products.

The values are reported as diagrams in Figure 2 in order to get a clearer visual representation of the cleaning effectiveness results. The effectiveness of the reference products (see Figure 2, ref. line 1 and ref. line 2) on the graph is an eye-guide, which should help in grouping the graffiti remover formulations: highly effective removal (ΔE ≥ 85%, 600 s) (above the line ref. 2—Nitro solvent) and poorly effective removal (ΔE ≤ 15%, 600 s) (below the line ref 1—petroleum ether). The area between the lines (ref. 1 and ref. 2), which is designated by the reference products, represents the satisfactory efficiency of paint removal from the surface but does not meet the primary goal of the production of an eco-friendly graffiti remover, which should be at least as efficient as the Nitro solvent (ΔE ≥ 85%, 600 s).

As seen in Figure 2, the removal of the paints from the support was achieved in all cases, with at least four or more of the selected eco-friendly graffiti removers being better than the Nitro solvent (MG-400-RO formulation no. 1, 8, 9, 19, 20; MG-400-SO formulation no. 1, 12, 14, 19; MG-400-UCO formulation no. 1, 8, 9, 18, 19, 20). Interestingly, no correlation seems to exist between the chemical nature of the paint polymeric binder and its removal efficiency.

Significantly, in certain cases, graffiti removers containing a similar level of green solvents behave very differently when they are mixed with water. The MG-400-SO-12, for instance, showed high effectiveness (ΔE > 85%, 600 s) in removing paints form the surface; however, it had a heterogeneous appearance. Obtaining a homogeneous appearance, however, would be more desirable, as it would indicate the higher stability of the tested formulation.

Nonetheless, the most effective formulation cannot easily be chosen by combining the effectiveness evaluation results with the information reported in Table S1. According to our results, compositions MG-400-RO formulation no. 8, 19, 20; MG-400-SO formulation no. 12, 14, 20; and MG-400-UCO formulation no. 1, 8, and 20 were able to most significantly and efficiently remove graffiti paint from surfaces. Therefore, optimization methods were used when choosing the best ecological formulation for graffiti removal in which such factors as the effectiveness of paint removal, residence time on the surface, physical properties, and water content in the formulation were taken into account.

### 2.5. Optimization of the Graffiti Remover Formulation

The functional characteristics of the different eco-friendly graffiti removers were predicted by using the reduced quadratic d-optimal design and the exploration of the resulting response surfaces. The influence of formulation composition (formulation no. (A)) and the type of plant oil employed in esterified derivatives synthesis (B)) on response factors Y_1_ (viscosity), Y_2_ (density), Y_3_ (runoff speed), and Y_4_ (effectiveness after 120 s) were studied. The experimental matrix of the employed d-optimal design with 30 randomized experimental runs and the corresponding values of the independent and dependent variables is presented in Supplementary Table S2.

**Figure 3:**
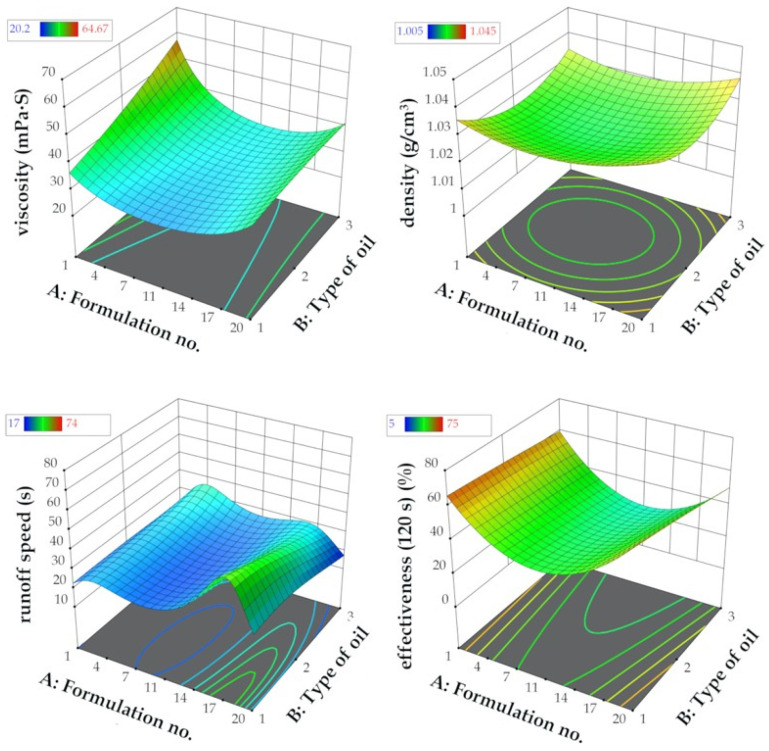
Graphical representation of the reduced quadratic d-optimal randomized design response surfaces for the dependent variables Y_1_ = viscosity, Y_2_ = density, Y_3_ = runoff speed, and Y_4_ = effectiveness (120 s) vs. the independent variables (no. of formulation (A), type of oil (B)).

The composition of each formulation based on plant oil type, i.e., MG-400-RO (rapeseed oil), MG-400-UCO (used cooking oil), MG-400-SO (sunflower oil), with the corresponding formulation numbers is presented in Table 3. The 3D response surface plot is the graphical representation of the regression equation used to investigate the existing interactions between the independent and dependent variables and to determine the optimal boundaries for the best graffiti removal formulation. The results obtained from the performed calculations and model fitting demonstrated that the optimal region for efficient graffiti removal is located on the external boundaries of 3D response surfaces, as shown in Figure 3. It represents the interactions between independent variables A and B and response factors Y_1_–Y_4_, understood as functionality measures.

To provide an example for such interaction, density above the value of 1.03 g/cm^3^ and viscosity below 40 mPa∙s resulted in noticeably increased runoff speed from flat surface (more than 25 s) and graffiti removal efficiency after 120 s from application (more than 70% of the surface was without previous paint contamination). Thus, a general conclusion supported by the results of the optimization is that regardless of the type of oil used in the formulation of a graffiti remover, the best composition was obtained in the case of the formulations no. 1 and no. 20. It turned out that water content that was too high (above 25%) as well as too low (less than 14%) in the graffiti removal formulation resulted in poor functional properties, i.e., runoff speeds less than 20 s and highly weakened effectiveness (less than 40%, even in prolonged application time). Therefore, the middle region of the response surfaces (Figure 3) might be considered as non-attractive and neglected in candidate selection. To conclude, six formulation candidates proposed by d-optimal model optimization with the corresponding predicted and actual values together with the desirability function measurements [49] are presented in Table 4.

The main goal of the optimization was to maximize the effectiveness of the graffiti removal formulation after the average contact time with the flat surface of 120 s. It was achieved for six formulations as mentioned above, which possessed functionality at the same or at higher level than the commercial reference removers (Ref. 1, petroleum ether—runoff time 5 s, effectiveness after 120 s 60%; Ref. 2, the Nitro solvent—runoff time 5 s, effectiveness after 120 s 65%). The following compositions: MG-400-RO-1 and MG-400-20; MG-400-UCO-1 and MG-400-UCO-20; MG-400-SO-1 and MG-400-SO-20, constituted by the oil ester content from 27.50% to 32.50% (*w*/*w*) and water from 15% up to 29% (*w*/*w*), resulted in good compromise between the cost and the quality of graffiti removal (effectiveness more than 40%). The d-optimal model fitting as well as the response regression equation are discussed in further sections. However, the best formulation turned out to be the MG-400-RO-20 (rapeseed oil PEG-8 ester 38.5% wt., ethyl lactate 30.0% wt., alkylpolyglucoside C8/C10 2.5% wt., water 14.0% wt.), which was later used in open-field testing, which is described further in the discussion section.

### 2.6. Attenuated Total Reflection—Fourier-Transform Infrared Spectroscopy (ATR FT-IR) Analysis

**Figure 4:**
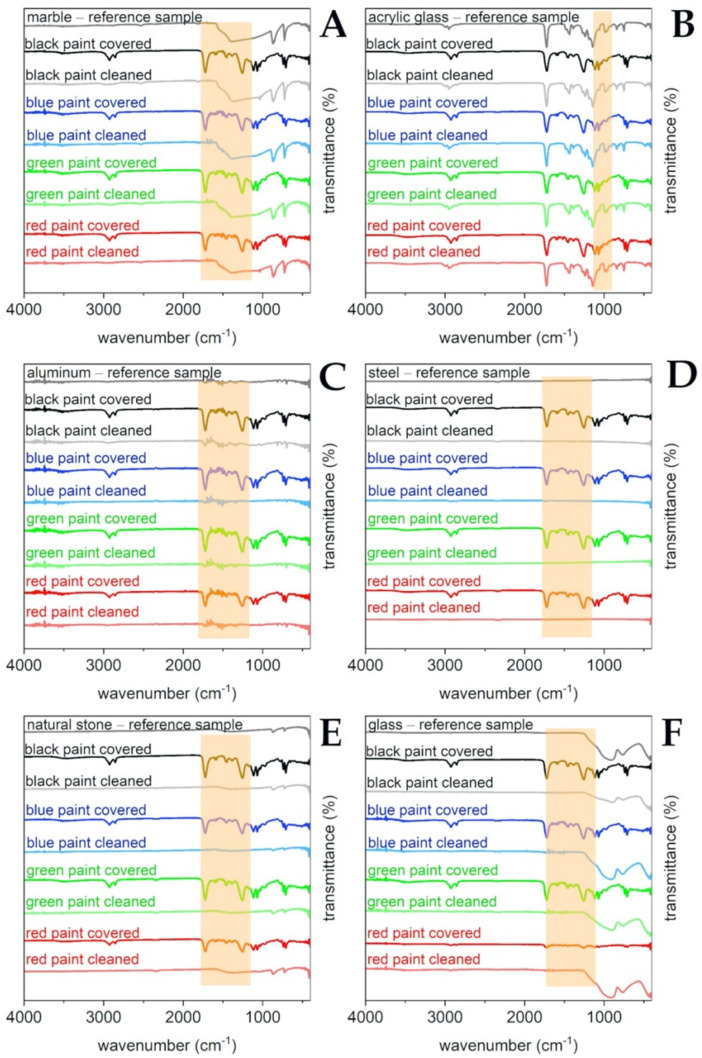
Results of selective laboratory paint removal tests on the selected substrates studied using ATR FT-IR spectral analysis. Reference materials and their samples covered with black, blue, green, and red paints together with the appropriate samples after cleaning procedures are shown in Figure 4A–F, respectively, in which: (**A**)—marble; (**B**)—acrylic glass; (**C**)—aluminum, (**D**)—steel; (**E**)—natural stone; (**F**)—glass.

Reference solid surfaces, i.e., marble, acrylic glass, aluminum, steel, natural stone, and glass, were subjected to ATR FT-IR analysis together with the samples covered with “Champion” paint sprays (black, blue, green, and red color) and after the laboratory cleaning procedure. Results of the analysis are presented in the Figure 4. The applied sprays are typical representatives of vinyl and acrylic based paints with diallyl phthalate pigments. Their IR spectra are considered to be relatively complicated due to the presence of resins, fillers, binders, and many other paint formulation components, which may produce overlapped signals [2,28].

The most characteristic signals derived from the paints that are present on the studied surfaces can be noticed in Figure 4. Bands at approximately 2950–2800 cm^−1^ are due to the presence of the symmetric and asymmetric stretching of the CH groups in the aliphatic chains. Moreover, a strong and narrow signal is produced by the carbonyl C=O stretching at approximately 1730 cm^−1^ as well as from the C-O stretching between 1020–1250 cm^−1^. Finally, signals at approximately 750 cm^−1^ are probably due to the presence of phthalate color pigments. Drawing conclusions from all of the studied references materials, the paint was successfully removed, and that can be supported by the absence of the above-mentioned characteristic signals on the cleaned solid surface spectra. Only in the case of acrylic glass (see Figure 4B) was it harder to observe the above given stretching signals since the studied substrates had similar structures to the used paints. However, the disappearance of the C-O stretching vibrations at 1020–1250 cm^−1^ is found to be clearly visible.

## 3. Discussion

The interests of green chemistry can be aligned with the industrial needs of having intensified processes with the use of raw materials from renewable sources or waste, high yields, and simplified downstream processing units. Thus, products containing green solvents derived from renewable materials have an increasing role in industrial cleaning technologies, and such research studies are highly desired. Based on the literature, an increasing trend is also observed in the replacement of traditional petrochemical solvents with green solvents such as ethyl lactate, propylene carbonate, or amphiphile-based nanostructured fluids (NSFs) [34,50]. The description “green solvent” implies a chemical that is benign to human health and the environment; therefore, it is worth noting that the esterified plant oil (RO, SO, UCO) with PEG 400 (denoted as Oil PEG-8 ester solvents) and ethyl lactate (EL) constitute ecologically friendly reactants—a new class of green solvents [33,50]. Both substances belong to the class of agrochemical solvents that can be derived from plant biomass. It should be noted that with an increasing awareness of sustainable development, green solvents are to be extensively explored nowadays as efficacious alternatives in place of toxic, volatile, harmful, flammable, and carcinogenic petrochemical solvents [51]. UCO use is especially very encouraging because it does not promote food competition [52]. Traditional solvents used as graffiti removers that are based on petrochemical solvents comprise a large part of the waste by-products from many branches of the chemical industry that cause various environmental and health problems [23]. At the same time, the so-called sugar surfactants do not persist in the environment after use, readily biodegrading into harmless compounds such as water and carbon dioxide [50,53].

The synthetic route of the Oil PEG-8 ester solvents meets all of the requirements that are necessary to develop new, green, and sustainable chemical technologies based on the application of products and processes that eliminate or reduce hazardous substances and residues. A profound example may be the fact that the discussed process prevents the storage of waste, including the used cooking oil as a substrate. It should be additionally underlined that according to the atom economy principle, in the case of all three oil substrates (RO, SO, and UCO), their incorporation in the appropriate final product (the respective Oil PEG-8 ester solvents) was maximized, which is reflected by the oil yield (see Table 1). Finally, the abovementioned oil-type solvents are considered as safer chemicals because they can easily be obtained from renewable resources by means of convenient catalytic processes that attain the required level of the pollution prevention hierarchy [45].

It has to be emphasized at this point that the surfactant presence in the graffiti removers is very important because decreasing the polymer (paint)/solid interfacial tension energetically favors film detachment from the solid surface, and a partial detachment of the paint from the surface is responsible for the first step of dewetting processes [33]. The specific role of surfactants in film dewetting comes from a thermodynamic and a kinetic standpoint; the surfactants differ in promoting detachment and dewetting. For example, in the presence of the oligooxyethylene-based nonionics C_9_–C_11_E6, the process is faster and more efficient than a standard anionic dodecyl sulfate SDS; however, both accelerate dewetting with respect to solvent/H_2_O mixtures. Finally, the efficiency of surfactants in terms of polymer detachment and dewetting is clearly boosted above the critical micelle concentration (CMC) values. From kinetic a aspect, the surfactants acquire a role in lowering the energy barrier, which prevents the paint from thermodynamically favored dewetting [54]. Many cleaning formulations require the presence of significant amounts of surfactants [31]. Nonionics revealing lower values of CMC in comparison to their ionic counterparts [55] make it possible to allow the application of smaller amounts of nonvolatile ingredients that might reside on the painting after the cleaning process. For many years, nonionic surfactants bearing a carbohydrate moiety as the hydrophilic part (so-called saccharide-based surfactants or sugar surfactants) have achieved increasing interest because of their profound surface and performance properties, their reduced environmental impact, and the fact that they can be synthesized from renewable sources [55,56,57,58]. It has been found that these materials exhibit similar general trends in surface-active behavior as those reported for polyoxyethylene-based nonionics [30,59]. Alkyl polyglycoside (APGs)—easily accessible from bioresources, non-toxic and degradable, proving good solubility in water and/or in organic solvents—has been found as a very amenable agent for the stabilization of nanostructured cleaning systems [2,60]. From the above reasoning, in the present study, APGs were selected as one of biodegradable components fulfilling the requirements of green and sustainable demands in an amount (2.5% *w*/*w*) similar to conventional nonionic surfactants.

**Figure 5:**
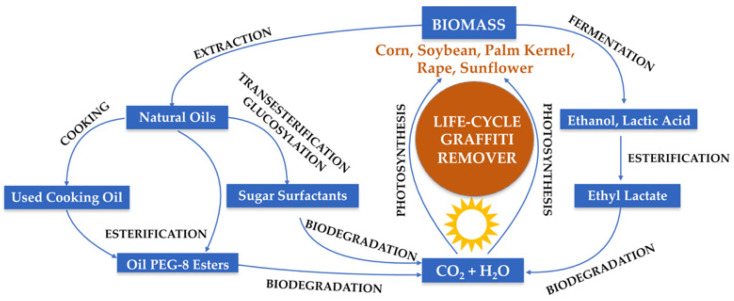
Lifecycle of all components of eco-friendly graffiti remover.

Figure 5 makes it clear that the production of eco-friendly graffiti removers is sustainable, taking into account the fact that plant biomass is used to generate natural alcohols, acids, and oils as valuable active substances for the purpose of creating innovative, safe, and environmentally friendly products. The formulation proposed in the present study is not only innovative, but it is also ecologically friendly, as the components are obtained from renewable and fully biodegradable resources. The considered product development life cycle of the graffiti remover, including product design, the processing of bio raw materials, products manufacturing, packaging, sales, and use as well as disposal after the whole process and additionally taking into account pollution prevention requirements, use of sustainable resources and energy, to reduce the impact of the production and consumption process on the environment, finally makes it possible for a graffiti removal product to meet the green attribute requirements. Additionally, it should also be emphasized that the environmentally safe graffiti remover formulated herein has a high potential for commercialization because it proves to possess very high efficiency in removing graffiti paint, and its production process complies with the principles of green chemistry.

**Figure 6:**
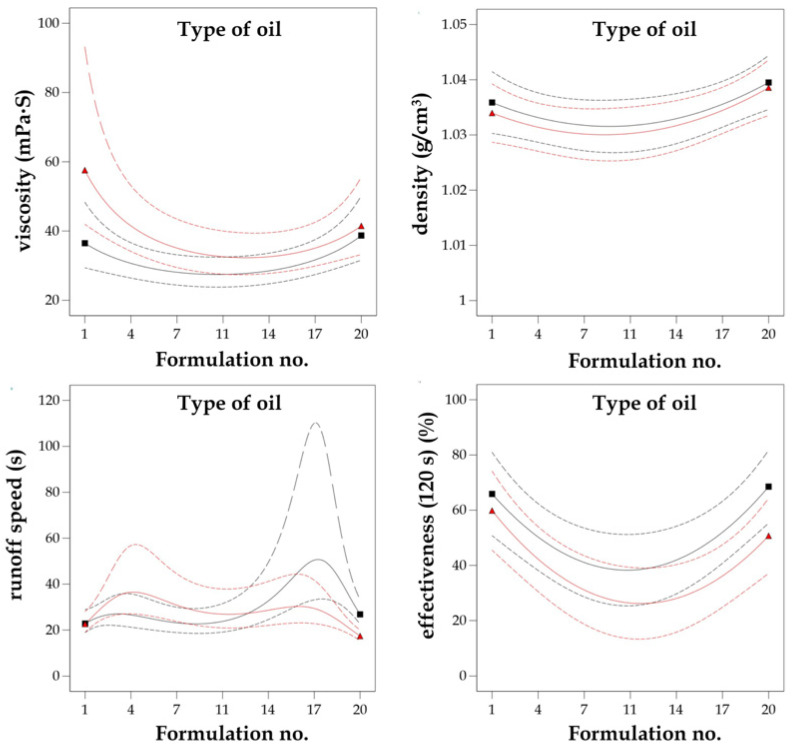
Interaction plots between independent variables the dependent variables (A = no. of formulation, B = type of oil) affecting the behavior of response variables (Y_1_ = viscosity, Y_2_ = density, Y_3_ = runoff speed, and Y_4_ = effectiveness (120 s)). The red line indicates sunflower oil, while black line corresponds to rapeseed oil.

The design of experiments (DoE) and quality by design (QbD) approaches based on statistical calculations are usually employed in formulation optimization. Analysis of variance (ANOVA) of the response surfaces estimated by d-optimal design for dependent variables Y_1_–Y_4_ indicated that the quadratic model exhibited the best fit in all cases, which is a typical solution in mixture designs in the literature [36,39,40,61]. The minor modifications for quadratic fitting were included for the viscosity (Y_1_) and the runoff speed (Y_3_) responses by implementing inverse linear regression in order to increase the robustness of the optimization model. The obtained best fit models had satisfactory statistical parameters, i.e., an insignificant lack of fit and highly matched R^2^ coefficients (both experimental and adjusted). For all four response factors, the *p*-values of the model fitting were less than 0.05 together with high F-values (higher than 3.00), implicating that all of the applied models were significant. The results of the ANOVA analysis are presented in Supplementary Table S3. For all of the studied dependent variables, no significant interaction effects were noticed for the formulation number (A) and the type of plant oil that was used (B). The predicted values of Y_1_–Y_4_ were mainly affected by the quadratic coefficient A^2^, where all of the obtained *p*-values were statistically significant in the ANOVA test. This clearly indicates that the composition formulation possesses the greatest influence on the functional properties of bio-based graffiti remover rather than the oil type included in the esterified derivatives, which is presented in Figure 6. As mentioned before, the percentage concentration of water (see Table 3) on the appropriate level as well as the amount of MG-400s esters will affect the effectiveness of the graffiti removal process to the highest extent. Moreover, as mentioned in the previous paragraphs (the results section), it should be emphasized that the appearance of the final formulation relied on the amount of water, and that usually, the cloudy or milky solutions were produced when the water content was above 25% *w*/*w*. To sum up, not only the effectiveness but also the appearance of the graffiti remover product is of special interest to future consumers.

The obtained quadratic regression equations from the d-optimal model fitted to the experimental values of the response factors were as follows:

**1/viscosity =** 0.0346 + 0.0012A − 0.0027B + 0.0019AB − 0.0094A^2^ + 0.0005B^2^ (1)

**density =** 1.02 + 0.0021A − 0.0007B + 0.0002AB − 0.0060A^2^ + 0.0084B^2^ (2)

**1/runoff speed =** 0.0472 − 0.0120A − 0.0024B + 0.0117AB − 0.0538A^2^ − 0.0059B^2^ (3)

**effectiveness (120 s) =** 32.75 − 1.63A − 5.96B − 2.94AB + 28.93A^2^ − 0.4304B^2^ (4)

which proves the relationship between the variables in the optimization procedure. In the case of the viscosity and density factors, the number of the formulation (A) contributes to the positive regression coefficients, and the type of plant oil used (B) contributes to the negative one. In the case of the quality measures of the graffiti removers, both the runoff speed and the effectiveness after 120 s show negative coefficients of parameters A and B, respectively. To conclude, both the exploration of the 3D response surfaces (Figure 3) as well as the interaction plots (Figure 6) together with candidate profiles followed by desirability function resulted in the selection of the MG-400-RO-20 formulation, which clearly reflects the main goals of optimization: cost-effective composition of graffiti remover formulation and maximized functionality, i.e., increased runoff time and effectiveness after 120 s from application when compared to the commercial reference products—petroleum ether and the Nitro solvent.

**Figure 7:**
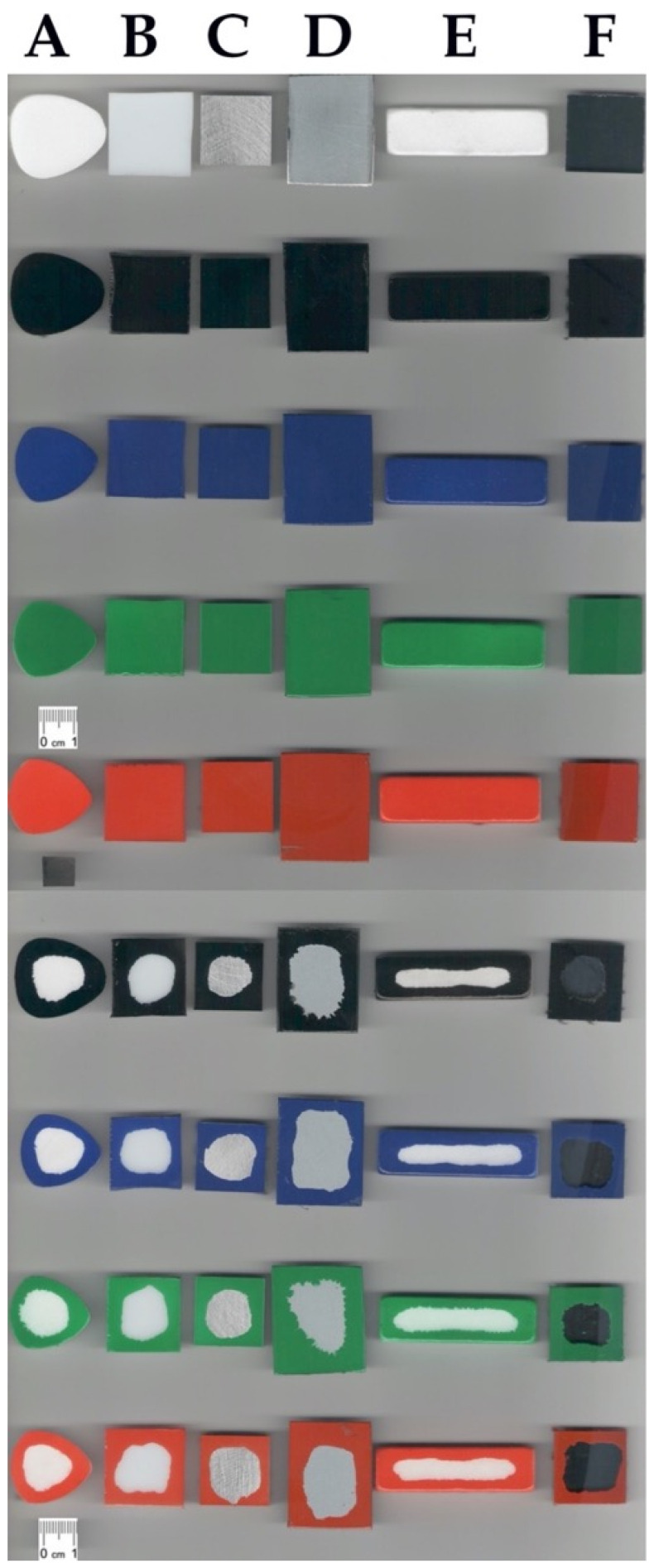
Complete set of samples used for the laboratory paint removal tests: (**A**) marble, (**B**) acrylic glass, (**C**) aluminum, (**D**) steel, (**E**) natural stone, (**F**) glass.

Laboratory selective graffiti paint removal tests were performed according to the laboratory cleaning evaluation described further in the Section 4.6 with the optimized formulation, i.e., MG-400-RO-20. Figure 7 reports the complete set of samples with the cleaning results. It was found that a single application of MG-400-RO-20 on the paint layer is very effective on metal, glass, aluminum, and acrylic glass solid surfaces. On the other hand, marble and natural stone materials required a double application of this formulation to completely remove paint from those porous substrates.

All of the reference surfaces were further characterized by means of the high-resolution pictures and the ATR FT-IR measurements. Figure 7 shows samples painted by black-, blue-, green-, and red-colored paints, respectively. All FT-IR analyses and the reported photographs are consistent with the conclusion that the paint layer was completely removed. From the FT-IR spectra (Figure 4), it can be noticed that the reflectance profiles of the analyzed substrate are similar before and after the cleaning process, while the spectrum of the paint color is significantly different from the cleaned substrate, proving that paint residues are either absent or undetectable within instrumental sensitivity. 

According to the results of efficiency tests, optimization, and laboratory cleaning assessment, the proposed eco-friendly graffiti remover formulation could be subjected to evaluation on a genuine graffiti painting during an outside-the-laboratory experiment [6].

**Figure 8:**
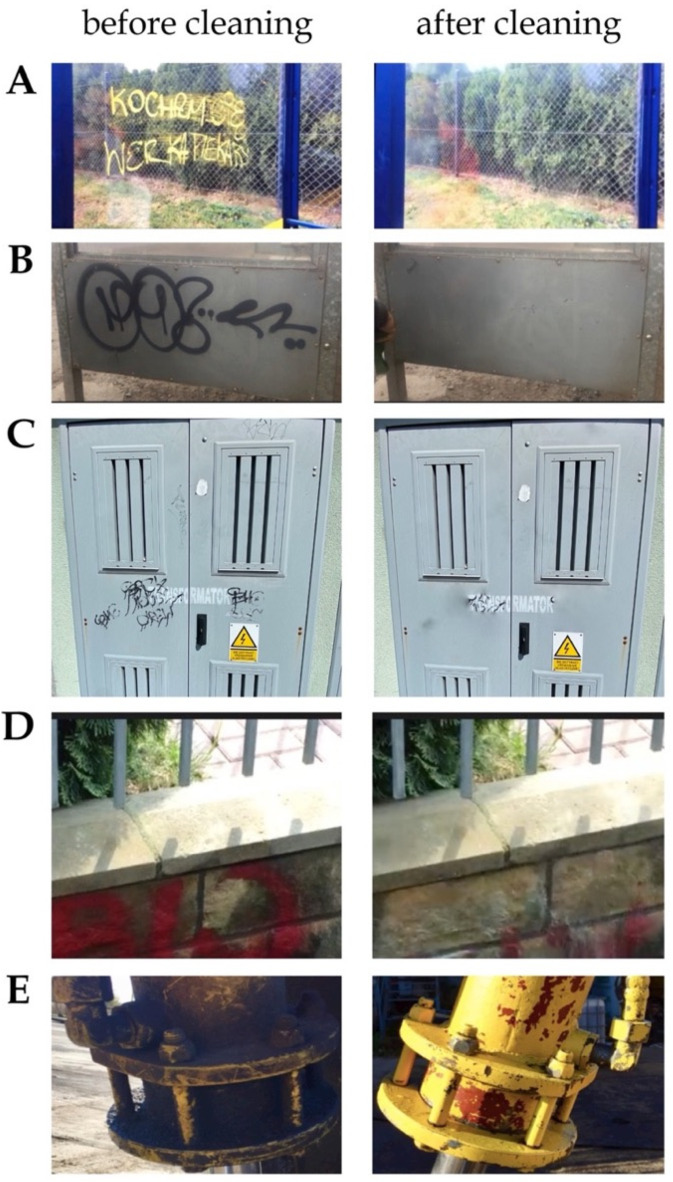
Different graffiti surfaces cleaned with eco-friendly graffiti remover MG-400-RO-20 in the city of Wrocław. (**A**). Glass surface at a bus stop. (**B**). Metal surface at a train stop. (**C**). Metal surface of a container. (**D**). Sandstone on a garden wall. (**E**). Metal surface of an excavator.

Therefore, as an example of a real graffiti cleaning case, the proposed eco-friendly graffiti remover formulation MG-400-RO-20 was tested in the removal of graffiti paint from different substrates in the city of Wrocław (as shown in Figure 8). The tests for removing graffiti paint from surfaces such as metal, glass, and sandstone in its natural condition were performed as follows: the graffiti remover was applied at a distance of 200 mm on test area with 600 s of initial contact of the product with the painted surface. After this time, a sponge was used to remove the maximum amount of graffiti possible. After graffiti removal, the surfaces were rinsed with water. As shown in Figure 8, old graffiti paints were completely removed from glass surfaces (see Figure 8A) and various metal surfaces (see Figure 8B,C,E), while slightly lower efficiency graffiti removal was achieved on the sandstone surface (see Figure 8D). The lower effectiveness of the eco-friendly graffiti remover in removing graffiti paint from stone surfaces may be associated with the much higher porosity of this type of substrate than that of glass or metal [10,11]. It is worth noting the complexity of graffiti removal resulting from the great number of variables at play: ageing of the paint, weathering, its chemical nature, roughness and porosity of the substrate, and others [6]. In fact, graffiti is hardly ever removed after it is created, i.e., most of the time, cleaning takes place after long environmental exposure. Consequently, the graffiti interacts with the environmental agents (e.g., rain and atmospheric pollutants) and also with the substrate. The graffiti paint may suffer physical and chemical alterations, which makes it more difficult to remove [6]. According to the study presented in this paper, when tested under natural conditions, eco-friendly graffiti remover formulation MG-400-RO-20 was found to be effective for the selective and controlled graffiti removal from different surfaces, proving that our developed and optimized formulation is an attractive and convenient alternative to the use of petrochemical organic solvents in graffiti paint cleaning.

## 4. Materials and Methods

### 4.1. Materials

Rapeseed oil and sunflower oil were purchased from P.P.H.U. ERJOX A. Mazur & J. Mazur (Błaszki, Poland), while the used cooking oil was purchased from Chemya (Poznań, Poland). Polyethylene glycol 400 (CAS Number: 25322-68-3) was purchased from Donauchem Polska (Poznań, Poland). Tetra-n-butyl orthotitanate (CAS number: 227-006-8) and Monobutyl tin tris-(2-ethylhexanoate) (CAS Number: 23850-94-4) were received from Sigma-Aldrich (Poznań, Poland). Ethyl lactate (Purasolv EL, Corbion) (CAS number: 687-47-8) was purchased from Envolab (Długołomice, Poland). Alkylpolyglucoside C8/C10 (APG) (CAS: Number: 68515-73-1) was purchased from OQEMA (Ozorków, Poland). Minclear SG36, Minclear NQ70 and Minclear NC150 (Diatomaceous earth) were a kind gift from Tolsa (Madrid, Spain). Ion exchange resins (Amberlyst A26, Amberlyst A46) were a kind gift from DuPont, (Warszawa, Poland). Champion spray paints were purchased form Champion Color Plus P. Lelito Sp. J. (Połchowo, Poland). Nitro solvent (mixture of toluene and acetone) and petroleum ether were purchased from Dragon Poland Sp. z o.o. (Skawina, Poland). Organic solvents, acids, and hydroxides were of analytical grade and were received from Avantor (Gliwice, Poland).

### 4.2. Preparation of Esterified Natural Oil or Used Cooking Oil with PEG 400

#### 4.2.1. Purification of Used Cooking Oil

The purification of the used cooking oil was conducted in a 2000 mL three-neck glass flask connected with a reflux condenser and a thermocouple probe. The mixture was agitated using a stainless steel stirrer. The glass flask was placed in a heated oil bath. Diatomaceous earth as a mixture of alkaline and acid adsorbents (1.0 g NQ70, 1.0 g NC155 and 1.0 g SG36) was added to the glass flask containing about 1000 g of used cooking oil heated up to the desired temperature of 70 °C. The mixture of oil and the diatomaceous earth was stirred for 60 min while being heated continuously. Afterwards, the diatomaceous earth was separated from the oil through filtration using two layers of filter paper. Next, the treated and used cooking oil underwent measurements of the acid value, the saponification value, the ester value, and the water content.

#### 4.2.2. Synthesis of Esterified Natural Oil or Used Cooking Oil with PEG 400 (Oil PEG-8 Ester Solvent)

The esterification/transesterification of the plant oils (i.e., rapeseed oil (RO), sunflower oil (SO), or used cooking oil (UCO)) was conducted in a 2000 mL Parr Temperature-Controlled Pressure Reactor. The mixture was agitated by using a stainless steel stirrer comprising a turbine. The reactor was heated by an external heating jacket. Monobutyltin tris(2-ethylhexanoate) and titanium(IV) butoxide were used as catalysts in this process. Catalysts were dissolved in PEG 400 before being poured into the reactor, which contained about 850–900 g of oil, and were heated up to a desired temperature, which was 70–80 °C. The reaction was kept at the desired temperature of 140–150 °C for 8 h. The molar ratio of the natural oil or the purified used cooking oil and the PEG 400 was 1:1.2 while the amount of Tin (Sn^4+^) and Titanium (Ti^4+^) catalyst in both was 150 ppm of the total amount mass reaction mixture. Reaction control measurements were performed through classic laboratory analysis (water content, acid value, saponification value, ester value) from samples taken every two hours. The synthesis of the Oil PEG-8 ester solvents was continued until the acid value of the reaction mixture was not higher than 0.05 mg KOH/g and until the saponification value was stabilized. After the reaction finished, the temperature of the Parr Temperature-Controlled Pressure Reactor was decreased to 70–80 °C, and the adsorbent mixture consisting of diatomaceous earth (1.0 g NC155, 1.0 NQ70) and anionic ion exchange resin (Amberlyst A26) and cationic ion exchange resin (Amberlyst A46) were added to the reactor. The contacting process of the post-reaction mixture with adsorbent mixture was conducted for 60 min at a temperature of 70–80 °C, while still having a mixture of adsorbents uniformly suspended in a solution of plant Oil PEG-8 ester solvent compounds in a stirred reactor. After the completion of the purification process, the product was filtrated at 70 °C using two layers of filter paper [62]. The composition of the eco-friendly graffiti removers, sample abbreviations, and their functional characteristics are provided in Table 3.

### 4.3. Preparation of Eco-Friendly Graffiti Remover Formulations

In order to obtain preparations for eco-friendly graffiti removers, formulations were prepared by mixing Oil PEG-8 ester solvent, ethyl lactate, alkylpolyglucoside C8/C10, and water in different proportions. All formulations were prepared by the cold blending (20 °C) of the three components, the Oil PEG-8 ester, the ethyl lactate, and the APGs. To avoid overheating, water was added last, taking into account the effect of heating up the formulation, which caused its excessive thickening, leading to the formation of a paste.

Formulation of the eco-friendly graffiti removers was followed by evaluating their macroscopic characteristics and physical properties. Organoleptic properties such as colour, physical appearance, and homogeneity were evaluated by visual perception immediately after preparation. Physical properties such as viscosity and density were measured using the automatic densimeter DA-640 Kyoto Electronic and the RST-50-2 Rheometer Brookfield with a Julabo heating-cooling system. The composition of the eco-friendly graffiti removers and the measured physical properties of the solutions are presented in Table 3.

### 4.4. The Speed of Runoff Graffiti Remover from Surface 

Plates made of acrylic glass (PMMA) were used for the tests. The plate was 500 × 200 × 10 mm. Groups of 5 acrylic glass samples were degreased with acetone and washed with demineralized water and dried (*n* = 300 samples). On each plate, in the upper part, an area of 100 × 100 mm was marked, to which the preparation was applied from a distance of 200 mm [10,11]. The speed of runoff graffiti remover from the surface was calculated as the time necessary to flow through the distance of 300 mm.

### 4.5. The Effectiveness Removing Graffiti Paints from the Flat Surface 

Plates made of acrylic glass (PMMA) were used for the tests. The plates were 200 × 200 × 10 mm. The samples were covered with selected black, blue, green, yellow, and red graffiti paints. These colors were chosen because they are commonly used by graffiti painters [8]. Plates made of acrylic glass (PMMA) were painted with a spray according to the method described by Sanmartin and Cappitelli [63]. The painted samples were left to air-dry under laboratory conditions (20 ± 5 °C and 40 ± 5% RH) for twenty-four hours. Groups of five PMMA samples were painted with each of the five graffiti paints with a single layer of paint (*n* = 300 samples), and an area of 100 × 100 mm was marked as the test area.

The eco-friendly graffiti remover was applied at a distance of 200 mm on the test area with 60, 120, 180, 300, and 600 s of initial contact of the product with the painted surface [64]. After this time, a sponge (made of emery stone and polyurethane foam, thickness 12 mm, size 120 × 100 mm) was applied in order to remove the maximum amount of graffiti. After graffiti removal, the acrylic glass surfaces were rinsed with demineralized water [10,11].

The effectiveness of removing graffiti paints was assessed by measuring the surface area from which the paint was removed. The effectiveness of cleaning with eco-friendly graffiti remover was calculated as follows:

ΔE (%) = (S_o_ − S_x_)/S_o_ × 100 (5)

where S_x_ is the area from which the paint was removed, and S_o_ is the initial area. 

### 4.6. Laboratory Cleaning Evaluation

Laboratory graffiti removal evaluation was done with the proposed eco-friendly graffiti remover formulation MG-400-RO-20. A small droplet of the discussed formulation, approximately 2–3 mL, was placed on the previously painted (24 h prior to cleaning procedure) examined flat surfaces, i.e., glass, metal, aluminum, acrylic glass, natural stone, and marble. The approximate size of tested material was 2–8 cm in width (see Figure 7). Subsequently, the swollen paint was gently cleaned off of the studied substrates with the abovementioned remover by a soft mechanical action using dry cotton swabs. Afterwards, the reference covered with paint and cleaned solid surfaces were photographed by means of high-resolution photography. Images were recorded perpendicularly to the photographed substrates, with a minimum resolution of 1200 dpi. 

### 4.7. Attenuated Total Reflection—Fourier-Transform Infrared Spectroscopy (ATR-FTIR) Analysis

Prior to Fourier transform infrared (FTIR) analysis, the spectra of the examined samples were recorded using IR Spirit spectrophotometer (Shimadzu, Japan) with an attenuated total reflectance (ATR) sampling accessory equipped with a diamond crystal (4000–400 cm^−1^, 64 scans, 2 cm^−1^ resolution, room temperature). The absorption spectra in the range 3800–700 cm^−1^ were analyzed by LabSolutions IR software (Schimadzu, Japan) by subtraction of the baseline and smoothing by reducing the noise from the water and the carbon dioxide.

### 4.8. Optimization of the Studied Safe Graffiti Remover Formulation by RSM

Response surface methodology was used to determine the optimal formulation of the green graffiti remover. A randomized reduced quadratic D-optimal design with a coordinate exchange subtype was developed by Design Expert Software (ver. 13.05.0, State-Ease, Inc., Minneapolis, MI, USA) [36,37]. A modified 2^k^ full factorial d-optimal design (presented in Supplementary Table S4) was used to optimize two significant factors, i.e., type of graffiti removal formulation (A) at 20 levels, and the plant oil used in the synthesis of esterified derivatives (B) at three levels ((−1) rapeseed oil; (0) used cooking oil; (+1) sunflower oil). In this study, 60 candidate experiments (Table 3) lead to the formation of a 30-run d-optimal experimental matrix, which was the best option possible in the use of the maximization criterion of the response factors. The response surface was used to explore the influence of the independent variables on the response factors (dependent variables). The physical properties, i.e., viscosity and density, as well as the efficiency of the graffiti remover, i.e., the runoff speed and the effectiveness on contaminated surfaces 120 s after application, were considered as the response factors (Y_1_), (Y_2_), (Y_3_) and (Y_4_), respectively. 

The following second-order polynomial equation based on the optimization design model represents the correlation between independent and dependent variables [36]:

Y_1_, Y_2_, Y_3_ or Y_4_ = β_0_ + β_1_A + β_2_B + β_1,2_AB + β_1,1_A^2^+ β_2,2_B^2^ (6)

where Y_1_–Y_4_ are the dependent variables and A and B are independent variables, respectively; β_0_ is an intercept term, β_1_ and β_2_ are linear coefficient, and β_1,2_ is an interaction coefficient, while β_1,1_ and β_2,2_ are the quadratic coefficients. 

The obtained regression model was evaluated by analysis of variance (ANOVA) and the corresponding statistical parameters, i.e., *p*- and F-values. The R^2^ coefficient was used to evaluate the quality of the optimization design model fitting to the experimental results. Finally, in order to determine the optimal formulation and the desirability of the eco-friendly graffiti remover and to explain the correlation between factors, the obtained polynomial equations for the Y_1_–Y_4_ response factors were presented in the form of a 3D response surface and X,Y plots [36,37].

## 5. Conclusions

In summary, three plant oil-PEG-8 ester solvents were synthesized via the direct esterification/transesterification of oils (i.e., rapeseed oil (RO), sunflower oil (SO), or used cooking oil (UCO)) with monobutyltin(IV) tris(2-ethylhexanoate) and titanium(IV) butoxide as the catalyst under mild conditions of the performed processing. By combining the organometallic tin (Sn^4+^) and titanium(Ti^4+^), a very efficient catalyst was obtained for the direct esterification of the free fatty acids contained in plant oils and for the transesterification of fatty triglycerides with polyethylene glycols (PEG400). Regardless of the type of the plant oils used in the synthesized process, the physical properties of the Oil-PEG8 ester solvents were similar to each other, which then served as new green solvents to compose environmentally friendly and safe graffiti removers with other green solvents such as ethyl lactate (EL), water, and alkylopolyglucoside as the surfactant. 

The most efficient formulation content was determined by optimization through the response surface methodology (RSM). There were two independent variables that were tested and resulted in the conclusion that the formulation composition possesses the greatest influence on the functionality of the graffiti remover. Finally, out of six candidates determined by the statistical calculations and the exploration of the 3D response surfaces combined with the desirability function based on response factors quality, one optimal product was chosen, i.e., MG-400-RO-20, which was based on the rapeseed oil PEG-8 ester. Before conducting the in situ cleaning tests, the abovementioned optimal formulation was subjected to a laboratory paint removal test on the various substrates, where its high effectiveness was confirmed. Concerning the effectiveness evaluation of the MG-400-RO-20 under the open-field conditions in the city of Wrocław in Poland, the results highlight the high and versatile effectiveness in the removal of paint from different types of surfaces i.e., glasses, metals, or sandstone.

Through the use of inexpensive and readily available biobased, biodegradable raw materials, the method of direct plant oil esterification/transesterification with polyethylene glycols shows the potential of a route of oil modification to enhance the group of green solvents as the ones that are dedicated to the cleaning of graffiti paints. Taking into consideration compliance with green standards (e.g., biodegradable alkylopolyglucoside-type surfactants and esterified plant oils), paired with their high effectiveness, the tested formulation has a high potential for its prospective use in protecting cultural heritage and keeping public areas clean while bearing in mind health and safety as well as in promoting green chemistry. As a final conclusion, we could select the most efficient formulations for better performing and safer cleaning systems by means of response surface methodology (RSM) optimization. In the future, more detailed physicochemical studies will need to be performed to evaluate the interaction of the Oil PEG-8 ester solvents and APG surfactants with the paint components that are required to be removed and the surface to be protected.

## Figures and Tables

**Table 1:** The esterification/transesterification process monitoring (fabrication of Oil PEG-8 ester solvents).

Samples Abbreviation	MG-400-RO	MG-400-SO	MG-400-UCO
type of oil	Rapeseed oil	Sunflower oil	Used cooking oil
% free fatty acid (% *w*/*w*)	1.02	0.51	1.78
% oil yield (% *w*/*w*)	98.98	99.49	98.22
feed molar ratio (oil: PEG-8)	1:1.2	1:1.2	1:1.2
mass oil (g)	850	850	900
mass PEG-8 (g)	480	480	480
Process Control Monitoring			
Time (h)	Acid value (mg KOH/g)		
0	1.28	0.64	3.26
2	0.62	0.24	1.25
4	0.21	0.09	0.13
6	0.05	0.04	0.06
8	0.02	0.02	0.03
Time (h)	Saponification value (mg KOH/g)		
0	131.02	123.35	174.78
2	130.40	123.11	173.53
4	130.19	123.02	173.40
6	130.14	122.98	173.34
8	130.12	122.96	173.31
Time (h)	Hydroxyl value (mg KOH/g)		
0	110.21	114.17	125.41
2	108.34	112.23	123.28
4	107.57	111.44	122.12
6	106.84	110.68	121.29
8	106.72	110.54	121.54
Time (h)	Water content (% *w*/*w*)		
0	0.213	0.127	0.371
2	0.015	0.015	0.016
4	0.012	0.011	0.012
6	0.010	0.010	0.010
8	0.009	0.008	0.009

**Table 2:** Physical properties of plant Oil PEG-8 ester solvents.

Samples Abbreviation	MG-400-RO	MG-400-SO	MG-400-UCO
Type of oil	Rapeseed oil	Sunflower oil	Used cooking oil
Physical state at 25 °C	Yellowish transparent liquid	Yellowish transparent liquid	Brownish transparent liquid
% free fatty acid (% *w*/*w*)	<0.01	<0.01	<0.01
M_n_, (M)	645	640	550
Acid value (mg KOH/g)	<0.01	<0.01	<0.01
Saponification value (mg KOH/g)	130.15	122.92	173.25
Hydroxyl value (mg KOH/g)	106.71	110.50	121.51
Water content (% *w*/*w*)	<0.01	<0.01	<0.01
Density (g/cm^3^) (25 °C)	1.1321	1.1442	1.1332
Viscosity (mPa·s) (25 °C)	97.42	105.42	98.36

**Table 3:** The compositions of the eco-friendly graffiti removers and their functional characteristics.

	**Controlled Variables of the Composition**				**Functional Characteristics**		
**Formulation 1** **(Symbols)**	**MG-400-RO (%*w*/*w*)**	**EL** **(%*w*/*w*)**	**APG** **(%*w*/*w*)**	**W** **(%*w*/*w*)**	**Viscosity (mPa·s)**	**Density (g/cm^3^)**	**Appearance**
MG-400-RO-1	38.50%	30.00%	2.50%	29.00%	40.76	1.0345	1
MG-400-RO-2	29.00%	30.00%	2.50%	38.50%	24.76	1.0362	3
MG-400-RO-3	48.50%	30.00%	2.50%	19.00%	40.59	1.0402	1
MG-400-RO-4	42.50%	22.50%	2.50%	32.50%	26.35	1.0331	1
MG-400-RO-5	38.50%	20.00%	2.50%	39.00%	29.39	1.0323	1
MG-400-RO-6	38.50%	10.00%	2.50%	49.00%	18.74	1.0251	4
MG-400-RO-7	28.50%	30.00%	2.50%	39.00%	21.36	1.0387	2
MG-400-RO-8	28.50%	40.00%	2.50%	29.00%	30.07	1.0451	2
MG-400-RO-9	43.50%	30.00%	2.50%	24.00%	41.96	1.0395	1
MG-400-RO-10	54.80%	42.70%	2.50%	0.00%	54.84	1.0380	2
MG-400-RO-11	25.00%	30.00%	2.50%	42.50%	20.20	1.0285	3
MG-400-RO-12	20.00%	30.00%	2.50%	47.50%	19.45	1.0273	3
MG-400-RO-13	57.50%	30.00%	2.50%	10.00%	41.15	1.0380	1
MG-400-RO-14	62.50%	30.00%	2.50%	5.00%	41.1	1.0401	1
MG-400-RO-15	38.50%	25.00%	2.50%	34.00%	36.42	1.0297	1
MG-400-RO-16	38.50%	15.00%	2.50%	44.00%	18.54	1.0224	4
MG-400-RO-17	38.50%	5.00%	2.50%	54.00%	20.21	1.0148	4
MG-400-RO-18	38.50%	35.00%	2.50%	24.00%	29.31	1.0363	1
MG-400-RO-19	38.50%	40.00%	2.50%	19.00%	28.74	1.0395	1
MG-400-RO-20	38.50%	45.00%	2.50%	14.00%	27.75	1.0420	1
**Formulation 2** **(Symbols)**	**MG-400-SO (%*w*/*w*)**	**EL** **(%*w/w*)**	**APG** **(%*w/w*)**	**W** **(%*w/w*)**	**Viscosity (mPa·s)**	**Density (g/cm^3^)**	**Appearance**
MG-400-SO-1	32.50%	50.00%	2.50%	15.00%	25.18	1.0345	2
MG-400-SO-2	47.50%	40.00%	2.50%	10.00%	32.71	1.0362	2
MG-400-SO-3	48.50%	30.00%	2.50%	19.00%	53.13	1.0331	2
MG-400-SO-4	42.50%	22.50%	2.50%	32.50%	45.99	1.0261	0
MG-400-SO-5	38.50%	20.00%	2.50%	39.00%	36.15	1.0253	0
MG-400-SO-6	38.50%	10.00%	2.50%	49.00%	24.01	1.0181	3
MG-400-SO-7	28.50%	30.00%	2.50%	39.00%	25.97	1.0316	0
MG-400-SO-8	28.50%	40.00%	2.50%	29.00%	25.22	1.0380	0
MG-400-SO-9	43.50%	30.00%	2.50%	24.00%	54.10	1.0324	0
MG-400-SO-10	54.80%	42.70%	2.50%	0.00%	64.67	1.0421	2
MG-400-SO-11	46.00%	30.00%	2.50%	21.50%	53.68	1.0300	1
MG-400-SO-12	22.50%	30.00%	2.50%	45.00%	25.86	1.0258	0
MG-400-SO-13	51.00%	30.00%	2.50%	16.50%	53.02	1.0311	2
MG-400-SO-14	40.00%	40.00%	2.50%	17.50%	49.55	1.0363	1
MG-400-SO-15	21.00%	40.00%	2.50%	36.50%	26.15	1.0326	0
MG-400-SO-16	38.50%	15.00%	2.50%	44.00%	30.1	1.0182	3
MG-400-SO-17	38.50%	25.00%	2.50%	34.00%	45.32	1.0251	0
MG-400-SO-18	38.50%	30.00%	2.50%	29.00%	54.45	1.0284	0
MG-400-SO-19	37.50%	50.00%	2.50%	10.00%	26.42	1.0423	2
MG-400-SO-20	27.50%	50.00%	2.50%	20.00%	24.32	1.0409	2
**Formulation 3** **(Symbols)**	**MG-400-UCO (%*w/w*)**	**EL** **(%*w/w*)**	**APG** **(%*w/w*)**	**W** **(%*w/w*)**	**Viscosity (mPa·s)**	**Density (g/cm^3^)**	**Appearance**
MG-400-UCO-1	38.50%	30.00%	2.50%	29.00%	46.32	1.0259	1
MG-400-UCO-2	29.00%	30.00%	2.50%	38.50%	27.51	1.0242	3
MG-400-UCO-3	48.50%	30.00%	2.50%	19.00%	47.75	1.0261	1
MG-400-UCO-4	42.90%	22.29%	2.50%	32.32%	41.78	1.0191	1
MG-400-UCO-5	38.50%	20.00%	2.50%	39.00%	32.65	1.0183	1
MG-400-UCO-6	38.50%	10.00%	2.50%	49.00%	21.54	1.0112	4
MG-400-UCO-7	28.50%	30.00%	2.50%	39.00%	23.47	1.0246	2
MG-400-UCO-8	28.50%	40.00%	2.50%	29.00%	32.69	1.0309	2
MG-400-UCO-9	43.50%	30.00%	2.50%	24.00%	48.82	1.0254	1
MG-400-UCO-10	54.80%	42.70%	2.50%	0.00%	58.34	1.0350	2
MG-400-UCO-11	25.00%	30.00%	2.50%	42.50%	23.11	1.0235	3
MG-400-UCO-12	20.00%	30.00%	2.50%	47.50%	22.47	1.0230	3
MG-400-UCO-13	57.50%	30.00%	2.50%	10.00%	47.29	1.0268	1
MG-400-UCO-14	62.50%	30.00%	2.50%	5.00%	47.18	1.0273	1
MG-400-UCO-15	38.50%	25.00%	2.50%	34.00%	40.21	1.0200	1
MG-400-UCO-16	38.50%	15.00%	2.50%	44.00%	20.87	1.0190	4
MG-400-UCO-17	38.50%	5.00%	2.50%	54.00%	24.31	1.0054	4
MG-400-UCO-18	38.50%	35.00%	2.50%	24.00%	33.45	1.0276	1
MG-400-UCO-19	38.50%	40.00%	2.50%	19.00%	31.79	1.0315	1
MG-400-UCO-20	38.50%	45.00%	2.50%	14.00%	29.53	1.0356	1

Abbreviations: MG-400-RO—esterified rapeseed oil with PEG 400, MG-400-UCO—esterified used cooking oil with PEG 400, MG-400-SO—esterified sunflower oil with PEG 400, EL—ethyl lactate, APG—alkylpolyglucoside C8/C10, W—water. Appearance: 0—non-stable, cloudy, heterogeneous, 1—stable, transparent, homogeneous, 2—stable, milky, homogeneous, 3—stable, white emulsion, homogeneous, 4—stable, white paste, homogeneous.

**Table 4:** Candidates for the optimal eco-friendly graffiti remover proposed by d-optimal model optimization based on desirability function and comparison of predicted and actual values.

No.	Formulation Number ^a^	Type of Oil	Viscosity (mPa∙s)		Density (g/cm^3^)		Runoff Speed (s)		Effectiveness (%)		Desirability
			P ^b^	A ^c^	P	A	P	A	P	A	
1	MG-400-RO-20	RO	38.7	37.8	1.04	1.04	26.8	26.0	68.5	75.0	0.907
2	MG-400-RO-1	RO	36.5	33.8	1.04	1.04	22.8	21.0	65.9	72.5	0.870
3	MG-400-UCO-1	UCO	45.7	36.3	1.03	1.03	23.4	20.0	63.3	75.0	0.833
4	MG-400-UCO-20	UCO	41.0	45.5	1.03	1.04	25.9	24.0	60.1	70.0	0.786
5	MG-400-SO-1	SO	57.6	48.2	1.03	1.03	22.6	20.0	59.9	65.0	0.784
6	MG-400-SO-20	SO	41.5	44.3	1.04	1.04	17.3	17.0	50.7	42.5	0.653

^a^ As presented in Table 3; ^b^ Value predicted by the d-optimal model; ^c^ Actual experimental value.

## Data Availability

Not applicable.

## Supplementary Materials

The following are available online. Table S1: The runoff speed of eco-friendly graffiti remover from the surface. Each data point represents the mean ± S.D., *n* = 5, Table S2: A reduced quadratic d-optimal design experimental matrix of two independent variables with their corresponding values and analyzed response factors Y_1_–Y_4_: viscosity, density, runoff speed, and effectiveness after 120 s, respectively, Table S3: ANOVA results for d-optimal randomized design quadratic model for dependent variables of graffiti remover formulations, Table S4: d-optimal randomized design with corresponding independent variables and their coded value levels for the preparation of graffiti remover formulation.

## Sample Availability

All samples are available from the authors upon request.
