# Supplementary material for: Formulation of Environmentally Safe Graffiti Remover Containing Esterified Plant Oils and Sugar Surfactant
Source: Molecules. 2021 Aug 3;26(15):4706. doi: 10.3390/molecules26154706 (PMC8347057; doi:10.3390/molecules26154706)
Supplement: Supplementary file 1 [file molecules-26-04706-s001.zip › molecules-1291792-supplementary.pdf]

SUPPLEMENTARY MATERIAL

Article

Formulation of Environmentally Safe Graffiti Remover Containing Esterified Plant Oils and Sugar Surfactant

Marcin Bartman [1], Sebastian Balicki [1] and Kazimiera A. Wilk [1,*]

[1] Department of Engineering and Technology of Chemical Processes, Wrocław University of Science and Technology, Wybrzeże Wyspiańskiego 27, 50-370 Wrocław, Poland; marcin.bartman@pwr.edu.pl (M.B.); sebastian.balicki@pwr.edu.pl (S.B.); kazimiera.wilk@pwr.edu.pl (K.A.W.)

* Correspondence: kazimiera.wilk@pwr.edu.pl; Tel. +48 71 320 2828: (K.A.W.)

Table of Contents

Table S1. The speed of runoff eco-friendly graffiti remover from the surface. Each data point represents the mean ± S.D., n = 5.

Table S2. A reduced quadratic D-Optimal design experimental matrix of two independent variables with their corresponding values and analysed response factors $Y_1 – Y_4$: viscosity, density, runoff speed and effectiveness after 120 s, respectively.

Table S3. ANOVA results for *D*-optimal randomized design quadratic model, for dependent variables of graffiti remover formulations.

Table S4. *D*-optimal randomized design with corresponding independent variables and their coded value levels, for the preparation of graffiti remover formulation.

Supplementary Tables

Table S1. The speed of runoff eco-friendly graffiti remover from the surface. Each data point represents the mean ± S.D., n = 5.

Formulation	Runoff Speed, (s) ± S.D.	Runoff Speed, (s) ± S.D.	Runoff Speed, (s) ± S.D.
Type of Oil PEG-8 ester	MG-400-RO	MG-400-SO	MG-400-UCO
1	21 ± 1	20 ± 1	20 ± 1
2	44 ± 2	25 ± 1	45 ± 2
3	35 ± 2	40 ± 2	34 ± 1
4	23 ± 1	28 ± 1	21 ± 1
5	20 ± 1	24 ± 1	19 ± 1
6	70 ± 3	100 ± 4	73 ± 3
7	29 ± 1	30 ± 2	31 ± 2
8	27 ± 2	33 ± 2	25 ± 1
9	21 ± 1	28 ± 1	24 ± 1
10	16 ± 1	26 ± 1	19 ± 1
11	22 ± 1	34 ± 2	25 ± 1
12	20 ± 1	25 ± 1	23 ± 1
13	35 ± 2	41 ± 2	35 ± 1
14	36 ± 2	27 ± 2	37 ± 2
15	21 ± 1	25 ± 1	20 ± 1
16	21 ± 1	105 ± 4	72 ± 3
17	70 ± 3	27 ± 1	74 ± 3
18	73 ± 3	28 ± 1	24 ± 1
19	26 ± 2	21 ± 1	74 ± 3
20	26 ± 2	17 ± 1	24 ± 1
**Commerial product**	**Runoff Speed (s) ± S.D.**		
Ref. 1. Extracion Gasoline	5 ± 1		
Ref. 2. Nitro Solvent	5 ± 1		

Table S2. A reduced quadratic D-Optimal design experimental matrix of two independent variables with their corresponding values and analysed response factors Y_1 – Y_4: viscosity, density, runoff speed and effectiveness after 120 s, respectively.

Run	Formulation no. (A)	Type of oil (B)	Y_1: viscosity (mPa·s)	Y_2: density (g/cm^3)	Y_3: Runoff speed (s)	Y_4: effectiveness (120 s) (%)
1	20	1	37.75	1.042	26	75
2	1	2	36.32	1.0259	20	75
3	11	1	20.2	1.0285	22	5
4	10	2	58.34	1.035	19	50
5	20	3	24.32	1.0409	17	42.5
6	1	3	48.18	1.0345	20	65
7	15	2	37.21	1.02	20	45
8	1	3	48.18	1.0345	20	65
9	13	1	41.15	1.038	35	35
10	1	1	33.76	1.0345	21	72.5
11	20	3	44.32	1.0409	17	42.5
12	1	1	33.76	1.0345	21	72.5
13	20	1	37.75	1.042	26	75

14	5	1	29.39	1.0323	20	45
15	7	3	25.97	1.0316	30	32.5
16	3	3	46.13	1.0331	40	35
17	20	2	45.53	1.0356	24	70
18	1	1	33.76	1.0345	21	72.5
19	15	3	26.15	1.0326	25	40
20	10	3	64.67	1.0421	26	40
21	17	1	20.21	1.0148	70	10
22	1	3	48.18	1.0345	20	65
23	20	2	45.53	1.0356	24	70
24	15	1	28.42	1.0297	21	47.5
25	1	2	36.32	1.0259	20	75
26	1	1	33.76	1.0345	21	72.5
27	12	2	22.47	1.023	23	5
28	12	3	25.86	1.0258	25	70
29	7	2	23.47	1.0246	31	40
30	1	3	48.18	1.0345	20	65

Table S3. ANOVA results for *D*-optimal randomized design quadratic model, for dependent variables of graffiti remover formulations.

Source	Sum of sq.	Term df	F-value	p-value
dependent variable: viscosity				
Model	0.0010	5	3.56	0.0089
A-Formulation	0.0010	1	0.7161	0.3880
B-Type of oil	0.0003	1	4.62	0.0374
AB	0.0001	1	1.54	0.2218
A^2	0.0006	1	10.87	0.0020
B^2	2.804E-06	1	0.0484	0.8269
Lack of fit	0.0024	30	5.25	0.0021
S.D. = 0.0076, Mean = 0.0292, R^2 = 0.2979, Adj. R^2 = 0.2143				
Mathematical correlation between response factor and independent variables:				
1/Viscosity = $0.0346 + 0.0012A - 0.0027B + 0.0019AB - 0.0094A^2 + 0.0005B^2$				
dependent variable: density				
Model	0.0014	5	6.07	0.0003
A-Formulation	0.0001	1	2.09	0.0961
B-Type of oil	0.0000	1	0.3973	0.5319
AB	1.360E-06	1	0.0299	0.8635
A^2	0.0003	1	5.70	0.0215
B^2	0.0007	1	15.40	0.0003
Lack of fit	0.0019	30	5.03	0.0032
S.D. = 0.0067, Mean = 1.03, R^2 = 0.4196, Adj. R^2 = 0.3505				
Mathematical correlation between response factor and independent variables:				
Density = $1.02 + 0.0021A - 0.0007B + 0.0002AB - 0.0060A^2 + 0.0084B^2$				
dependent variable: runoff speed				
Model	0.0030	11	3.99	0.0008
A-Formulation	0.0002	1	3.14	0.0848
B-Type of oil	0.0000	1	0.6433	0.4278
AB	0.0002	1	2.63	0.1135
A^2	0.0007	1	10.33	0.0028

B^2	0.0001	1	1.54	0.2225
Lack of fit	0.0024	24	5.25	0.0005
S.D. = 0.0082, Mean = 0.0418, R^2 = 0.5495, Adj. R^2 = 0.4118				
Mathematical correlation between response factor and independent variables:				
1/runoff speed = $0.0472 - 0.0120A - 0.0024B + 0.0117AB - 0.0538A^2 - 0.0059B^2$				
dependent variable: effectiveness (120 s)				
Model	7692.82	5	4.64	0.0019
A-Formulation	82.52	1	0.2486	0.6206
B-Type of oil	1260.48	1	3.80	0.0580
AB	206.22	1	0.6214	0.4350
A^2	6012.43	1	18.12	0.0001
B^2	1.84	1	0.0055	0.9410
Lack of fit	13938.93	30	4.75	0.0032
S.D. = 18.22, Mean = 50.78, R^2 = 0.3556 Adj. R^2 = 0.2789				
Mathematical correlation between response factor and independent variables:				
effectiveness (120 s) = $32.75 – 1.63A – 5.96B - 2.94AB + 28.93A^2 - 0.4304B^2$				

Table S4. *D*-optimal randomized design with corresponding independent variables and their coded value levels, for the preparation of graffiti remover formulation.

independent variable	number of levels	value levels	
		**low value**	**high value**
A: formulation no.	20	No. 1	No. 20
B: type of oil	3	1 (rapeseed)	3 (sunflower)
